# The influence of spatial and temporal discontinuities of forest habitats on the current presence of flightless saproxylic beetles

**DOI:** 10.1371/journal.pone.0197847

**Published:** 2018-05-17

**Authors:** Eugénie Cateau, Pierre-Alexis Herrault, David Sheeren, Sylvie Ladet, Hervé Brustel

**Affiliations:** 1 UMR 1201 DYNAFOR, INPT-EI Purpan, University of Toulouse, Toulouse, France; 2 UMR 1201 DYNAFOR, INP-ENSAT, University of Toulouse, Toulouse, France; 3 UMR 1201 DYNAFOR, INRA, Toulouse, France; Università degli Studi di Napoli Federico II, ITALY

## Abstract

Flightless saproxylic beetles were selected in order to study the impact of temporal and spatial discontinuity of forests. They were chosen because: (1) they are unable to fly, making them dispersal-limited species, (2) they have a saproxylic diet, which means they are closely linked to the forest, and (3), they have rarely been studied. Forest temporal continuity was expected to be the main factor explaining the presence of these species, modulated by the past and present amount of forest in the surrounding landscape. Twenty-seven forests, distributed into three zones, were sampled in southwestern France. Flightless saproxylic beetles were surveyed using a Winkler extractor and a Berlese funnel. Their presence/absence were modelled using generalised linear mixed models, with zone variable as random effect. Two species showed significant *zone* effect and were only or more present in the zone with the highest present forest amount in a 0.5 km radius. In the model that converged, the only selected variable was the past amount of forest in the landscape. The size of the forest, the presence of dead wood and the forest temporal continuity were not included in this model. The importance of the amount of forest in the landscape supports the hypothesis that dispersal-limited species are affected by landscape characteristics. This study demonstrates an important link between the presence of *Dienerella clathrata* and the amount of forest in the past, which led to an indicator species analysis being performed.

## Introduction

The temporal and spatial discontinuity of habitat in the landscape presents a major threat to biodiversity [1–3]. Reduction in habitat (by habitat loss and fragmentation) is harmful for species that are dependent on this habitat [4–6]. Habitat loss is a decrease in the habitat area through is destruction [5]. Fragmentation is the separation of habitats although, the amount remains the same [5]. The impacts of habitat loss and fragmentation differ between species or groups of species [5]. For specialist species living in only one habitat, in this case forests, habitat loss and fragmentation have negative impact on species abundance [7,8]. Habitat loss has greater negative impacts on biodiversity than fragmentation does [1,5].

Temporal discontinuity of forest habitats has been caused by past deforestation or afforestation event(s). Here, two types of forest were considered: continuous forest, i.e. an area in which the forest habitat has been present for a long time, namely, ancient forests, and discontinuous forest, i.e. an area that has been deforested or afforested in the past, namely recent forests [9]. Differences in species composition have been reported in the two types of temporal continuity, especially that of herbaceous species [10,11]. Depending to a certain extent on their colonisation ability, species are able to recolonise newly afforested areas, i.e. recent forest, at different speeds [12]. The resilience of species to temporal and/or spatial discontinuity therefore depends on their dispersal ability.

Flightless saproxylic litter beetles (FSLB) are species with dispersal limitation due to their inability to fly and their small size (less than 5 mm). They are an optimal and original biological model for studying the impact of human disturbance on dispersal-limited species in space and over time.

FSLB differ from other known saproxylic beetles in three main aspects: (1) their motion, (2) their phenology and adult lifespan and (3) the type of wood in which they develop.

FSLB are different from species that are able to fly because they have a morphological dispersal limitation [13]. Their small size (< 5 mm) also makes FSLB different from larger flightless saproxylic beetles such as *Morimus asper* (Sulzer, 1776), as their small legs mean they are slower moving.

FSLB can be found at adult stage in the litter throughout the year. For most of the species, there is no monthly difference in their abundance, other FSLB show variations in their abundance but are always present [14]. Some of them have lived in their adult stage more than one year in captivity [15]. These breeds and their phenology strongly suggest that these species have an adult lifespan of one year (or more). In contrast, the majority of other saproxylic beetles live at the adult stage for just few days or weeks, in warm weather [16].

In the literature, most of the studied saproxylic beetles develop in dead wood that has certain characteristics: it is large and/or at a certain stage of decomposition and/or abundant in the stand [17–21]. In contrast, neither the quality nor quantity of dead wood can explain the distribution of FSLB [21,22], suggesting that these species develop in thin branches, which is consistent with their small size [20].

These three major points (motion, adult lifespan and diet) show how FSLB are different from what is known about other saproxylic beetles. It may suggest that the presence of these species presence could be driven by factors that differ from what is expected for saproxylic beetles (especially the quantity and quality of dead wood), and thus, why the study of them is important.

FSLB have been the subject of very few studies [21,23–25] and a sampling method dedicated to this group has only recently been published [14].

Recent literature focusing on this group consists of two main articles that have different results [23,24]. In [23], the author aimed “to test the frequency of relict species in relation to historical and current woodland size”. Five *Cryptorhynchina*e and one *Molytinae* species were studied, sampled by litter sieving in a highly fragmented landscape. Twenty-seven individuals were collected exclusively in 15 ancient (out of 29) forests, or in areas adjacent to ancient forests. The author concluded that these species are “relict species of an ancient woodlot in north western Germany”. In [24], the study investigated the dependence on continuous forests in ancient forests conditions. Seventy individuals of six species (*Cryptorhynchinae* and *Molytinae)* were collected in a natural park, of which three were similar to those found in [23]. As some individuals were caught in recent but old-growth forest, Horak et al. concluded that flightless saproxylic beetles “can reappear in secondary woodlands if [forests] reach their optimal structure”.

In Buse’s study [23], FSLB appear unable to recolonise recent forests, whereas in the study of Horak et al. [24], the same species are apparently able to recolonise such forests. One possible explanation for these contrasting results is the difference in the structure of the landscape surrounding the surveyed forest. In the highly forested landscape studied by Horak et al. [24], recolonization by FSLB may be easier than in Buse’s highly fragmented landscape. In other words, the amount of forest in surrounding area may affect FSLB’s distribution pattern, as already shown for other taxonomic groups [5,26,27].

In the present study, the species distribution of FSLB was investigated by combining the effects of temporal and spatial discontinuity. The relative importance of forest area, forest temporal continuity, current and past forest amounts in the landscape, on the presence/absence of these dispersal limited species was assessed. It was expected that:

- FSLB are less present or absent in recent forests because of their limited dispersal [23].
- large forest are richer than smaller ones [28].
- the amount of dead wood is of only minor importance for these species, even if they are saproxylic [21,22].
- the past and present relative amount of forest in the surrounding landscape has an effect on the presence/absence of these species, since habitat amount is considered a major threat to biodiversity, especially for dispersal limited species [5].

Thus, is was expected that the temporal continuity of the forest would be the main factor explaining the presence of FSLB, modulated by past and present amounts of forest in the landscape.

## Material and methods

### Study design

The study site is located in southwestern France [29,30], in the Long-Term Social-Ecological Research (LTSER) platform “Vallées et Coteaux de Gascogne” (43°26’02.08”N; 1°05’12.33”E). This high region (200–400 m) on the Pyrenees piedmont is about 60 km south-west of the city of Toulouse. The climate is temperate, with oceanic and Mediterranean influences. It is a rural landscape, consisting of cultivated fields and some grassland. Forest amount for around 20% of the landscape, but is highly fragmented [27,31]. The most frequent tree species are sessile oak (Q*uercus petraea* Mattus) and pedunculate oak (*Quercus robur* L.). All the forests are oak dominated and managed, mainly by coppicing with fine-scale logging events (< 1 ha).

The sampling plan in this study was based on two factors: the temporal continuity of the forest and the current extent of the forest.

The temporal continuity of a forest describes the time since a given area was first classified as a forest. In France, a historical map dating from 1850 enabled two types of forest to be defined: *ancient* for a forest that existed in 1850 and *recent* for a forest in a place that was not covered by a forest in 1850 and consequently appeared between 1850 and the present day [9]. For the ancient forest, the temporal continuity of the forest state between 1850 and 2010 was verified using aerial photographs taken between 1942 and 1948. Since forest cover in France has increased over the past two centuries, forest continuity was assumed for the studied period if a forest was present on these three dates [9].

**Fig 1:**
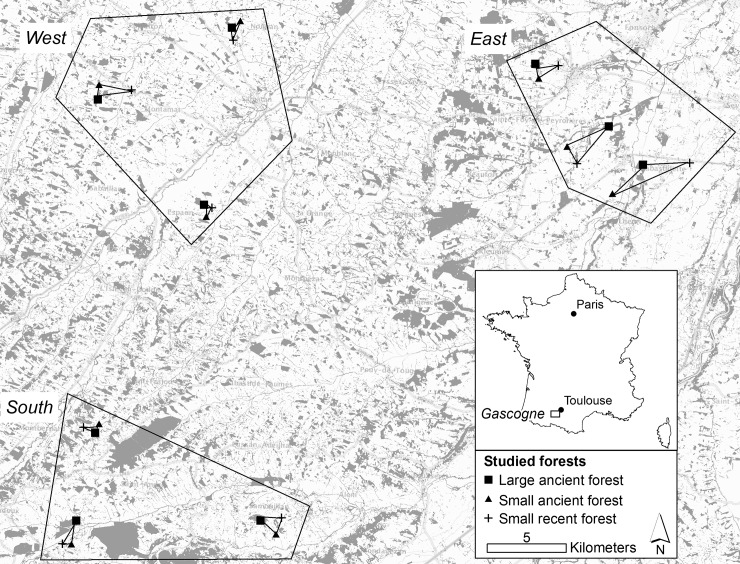
Sampling plan: 27 forests were sampled in a highly fragmented region. Grey indicates forest in the landscape.

Forests in the study region are usually small (average area of 4 ha) [31]. Two classes of forest were considered: *small* forests (< 3 ha) and *large* forests (> 10 ha). A total of 27 forests were sampled (Fig 1) and classified into three groups: 9 *large ancient* forests (> 10 ha, continuously wooded since 1850), 9 *small ancient* forests (< 3 ha, continuously wooded since 1850) and 9 *small recent* forests (< 3 ha, constituted after 1850). There is no large recent forest in the study area [31]. All the forest were privates and the owners gave permission for the study to be conducted on their sites.

The forests sampled were distributed into three zones: east, west and south. The three zones consisted of three groups of large ancient / small ancient / small recent forest.

### Spatial, historical and structural characteristics of the forest samples

The map of recent forests came from the national topographic vector database (BDTopo) produced by France’s National Institute of Geographic and Forest Information (IGN). BDTopo is typically defined to make maps on a 1:25,000 scale. The forest layer in the study area date from 2010. This layer was used to estimate the current area of each forest [32].

The amount of forest habitat was considered by calculating the forest density in the area surrounding each sampled forest (i.e. the percentage of forest cover including the focal forest) [33]. This variable was computed on two spatial levels (within a 0.5 and a 1 km radius) and on two dates (1850 and 2010). Forest within a radius of 0.5 km and 1 km from the centre of a sampled stand were assumed to provide appropriate habitats for FSLB [34].

The temporal continuity of the forest (ancient vs. recent) and the extent of past forests were derived from the French historical '*Etat Major*' colour map (scale 1:40,000) dating from the middle of the 19^th^ century [9,30,32]. The same spatial areas as those selected for the present forests were used, i.e. areas with a radius of 0.5 and 1 km.

To assess the importance of available dead wood for the species in this study, three variables were measured in three fixed-area plots within a 20 m radius located in a one hectare sample area within the forests; this was the entomological sampling plot, in which the beetles were surveyed. The three variables were: (1) the volume of standing dead trees (diameter at breast height > 7.5 cm), (2) the volume of lying deadwood (diameter > 2.5 cm) using the line intersect sampling (LIS) method [35] and (3) the number of tree species (i.e. taxonomic diversity).

### Species survey

Beetles were sampled in the 27 forests in February 2014 using the litter sieving method [14,23,36]. In each sampled forest, one homogeneous hectare in the center of the forest was selected to constitute the entomological sampling plot. In each plot, 10 trees representing the tree diversity were selected by the same person and adjacent litter sieved using a Winkler sieve with a 5 mm mesh. One litre of sieved litter was collected per tree, making ten litres per stand. The number of trees sampled and the litter volume are sufficient to collect 97.2% of the FSLB present, even in rich sites [14]. Arthropods were extracted from the litter using a Berlese extractor [14]. Species were sent to a specialist for identification by morphological analysis. Only flightless (without wings or with knitted elytra) saproxylic litter beetles were kept and included in the dataset.

### Statistical analysis

Ecological differences between the three zones (east, west and south) were tested by means of the Kruskal-Wallis test for each variable: Forest_amount 2010_0.5, Forest_amount 2010_1, Forest_amount 1850_0.5, Forest_amount 1850_1, Area 2010, STW, CWD, ITS, Histo_continuity (Table 1).

**Table 1:** GLM tested to explain the presence-absence of the four FSLB species recorded in a fragmented forest landscape.

Explanatory variables	Description	
*Model 0*.*5* with current area, past and present amount of habitat within a 0.5 km buffer radius, structural heterogeneity variables and historical continuity		
Model form: E(y) = β_0_ + β_1_+ β_2_0.5_+ β_3_0.5_+ β_4_+ β_5_+ β_6_+ β_7_+(1|β_8_)		
Area 2010	β_1_	Present area of woodland (topographic database 2010) (ha)
Forest_amount 2010_0.5	B_2_0.5_	Present forest amount in a zone with a 0.5 km radius (topographic database 2010) (%)
Forest_amount 1850_0.5	B_3_0.5_	Past forest amount in a zone with a 0.5 km radius (historical map of 1850) (%)
STW	β_4_	Volume of large standing deadwood per forest (m^3^/ha)
CWD	β_5_	Volume of coarse woody debris per hectare (m^3^/ha)
ITS	β_6_	Number of indigenous tree species per hectare
Histo_continuity	β_7_	Historical forest continuity over time (present in 1850 = ancient; recorded after 1850 = recent)
Zone (random effect)	β_8_	Zone (south east or west) in which the forest is located
*Model 1*.*0* with current area, past and present amount of habitat within a 1 km buffer radius, structural heterogeneity variables and historical continuity		
Model form: E(y) = β_0_ + β_1_+ β_2_1_+ β_3_1_+ β_4_+ β_5_+ β_6_+ β_7_+(1|β_8_)		
Area 2010	β_1_	Present area of woodland (topographic database 2010) (ha)
Forest_amount 2010_1	β_2_1_	Present forest amount in a zone with a 1 km radius (topographic database 2010) (%)
Forest_amount 1850_1	β_3_1_	Past forest amount in a buffer zone with a 1 km radius (historical map of 1850) (%)
STW	β_4_	Volume of large standing deadwood per forest (m^3^/ha)
CWD	β_5_	Volume of coarse woody debris per hectare (m^3^/ha)
ITS	β_6_	Number of indigenous tree species per hectare
Histo_continuity	β_7_	Historical forest continuity over time (present in 1850 = ancient; recorded after 1850 = recent)
Zone (random effect)	β_8_	Zone (south east or west) in which the forest is located

Generalised linear models (GLM) were used to link the presence-absence of species and the explanatory variables related to the forest characteristics. All the analyses were performed with R software 3.1.0 [37], the “stats” package [37] and the lme4 package [38]. Binomial distribution was used to fit the models based on the presence-absence surveys. The spatial effect of the zone was taken into account through the random effect in the model.

Since multicollinearity of predictors may inflate the variance of regression parameters, checks were undertaken to verify that the variables were not highly correlated (│r│< 0.7; [39]). The number of nodes in the quadrature formula chosen was 15. Marginal and conditional R^2^ were calculated. The variance of random effect was the main factor in model selection, then R^2^ led to selection of the best model.

Two different models including the non-correlated spatial, historical and structural forest variables were investigated (Table 1). The first was based on the forest habitat amount based on the 0.5-km buffer radius (*model 0*.*5*) and the second was based on the 1 km buffer radius (*model 1*.*0*). The two models were run for the four FSLB species recorded in this study and enough abundant (*Acalles misellus*, *Anchonidium unguiculare*, *Dienerella clathrata* and *Langelandia anophtalma)*

## Results

### Forest characteristics with their surrounding area

The forests investigated in this study are representative of small privately owned forests in southwestern France, with a mean total dead wood volume of 24.33 m^3^/ha, a tree species richness of 3.44 per forest, and an area ranging from 0.7 to 82 ha (mean 10 ha). The proportion of forest in the area surrounding the sampled forests was approximately 19%, demonstrating an increase since 1850, when the proportion of forest was 16%.

High correlations (│r│> 0.7) between variables were found for (1) the amount of ancient forest within the 0.5 km buffer radius area with the one based on the 1-km radius and (2) the amount of current forest for both areas. These correlations explain why two different models were built. All the variables integrated in the models had a correlation < 0.7, as recommended.

### Ecological differences between the three zones

**Fig 2:**
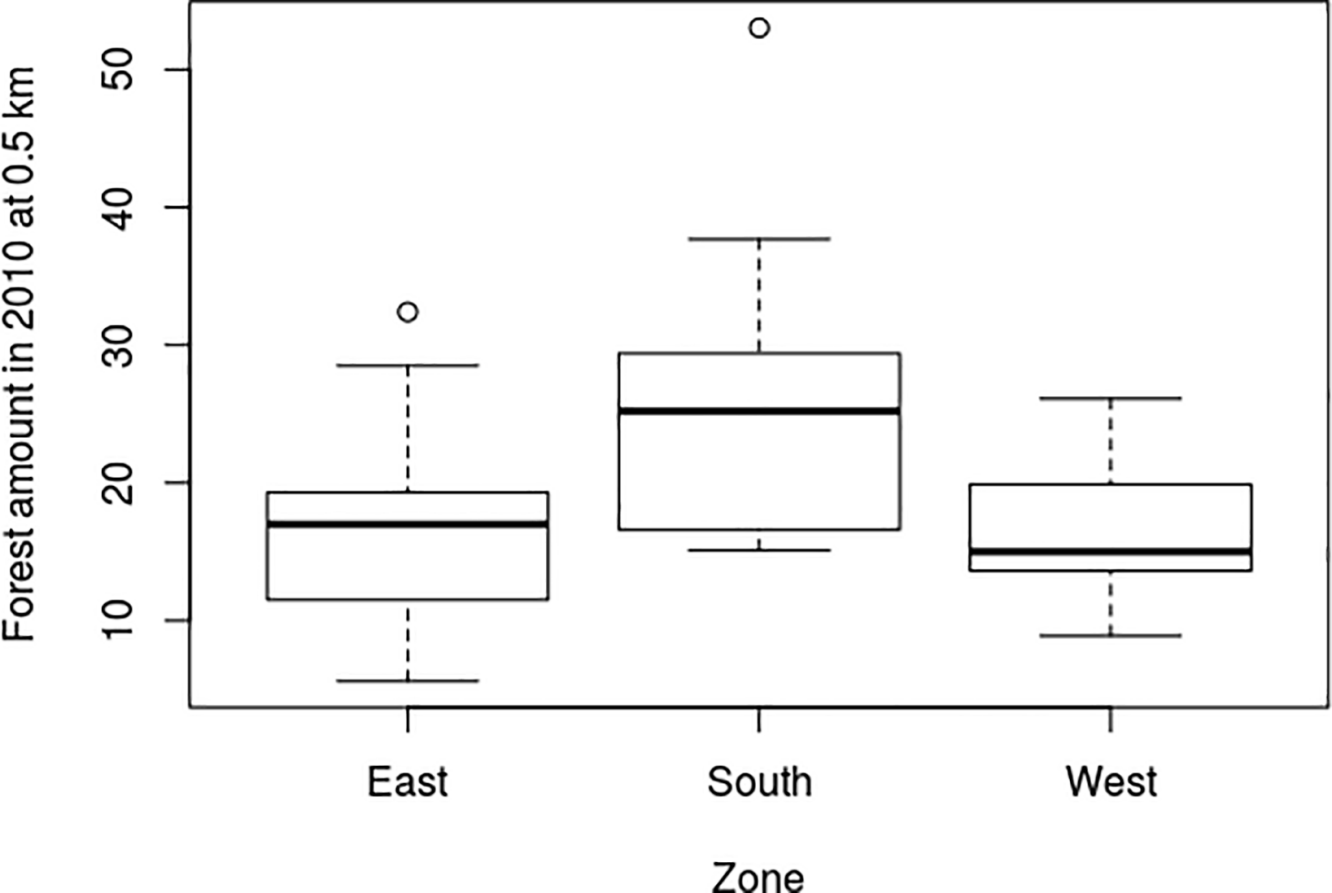
Forest amount in 2010 within a 0.5 km radius in the three zones.

There was no significant difference between the three zones for any of the tested variables at a 0.05 significance level (Kruskal Wallis test, p-value >0.05). At a 0.1 significance level, only the Forest_amount 2010_0.5 showed significant differences between the zones, with a p-value of 0.067. The *south*’s zone had a larger amount of forest than the *east* an *west* zones (Fig 2).

### Species distribution

Five species of FSLB were found (three *Curculionidae*, one *Latridiidae* and one *Zoopheridae*): *A*. *misellus*, *A*. *unguiculare*, *D*. *clathrata*, *L*. *anophtalma* and *Ruteria Hypocrita*.

Since only one individual of *R*. *Hypocrita* was collected, no statistical analysis was carried out on this species and it is disregarded in the rest of this paper.

Sixty-one *A*. *misellus* individuals were found in 10 of the sampled forests. Seven of these forests were situated in the *south* zone, two in the *east* zone and one in the *west* zone. GLMM showed a significant effect of the zone (Table 2, variance component of zone variable > 0). The three forests in the east and west zones where the species was found have a forest amount in 2010 with a 0.5 km radius above 19.2%, which was the seconds and the third highest values for these zones. The species was present in six ancient forests and four recent forests.

**Table 2:** Results of the GLMM; NA: Not applicable.

	*A*.*misellus*	*A*.*unguiculare*	*D*.*clathrata*	*L*.*anopthalma*
Model 0.5				
AIC	33.4	25.6	39.5	45.9
Fixed effect (estimate, with 95% confidence interval)				
Intercept	-7.45	-	-	-
Area 2010	-	-	-	-
Forest_amount 2010_0.5	0.68	-	-	-
Forest_amount 1850_0.5	-0.41	-	-	-
STW	0.14	-	-	-
CWD	-	-	-	-
ITS	-	-	-	-
Histo_continuity	-	-	-	-
Random effect (variance component)				
Zone	0	192,5	0	0
R^2^ marginal	0.93	NA	NA	NA
R^2^ conditional	0.93	NA	NA	NA
Model 1				
AIC	42.7	27.0	34.5	43.2
Fixed effect (estimate, with 95% confidence interval)				
Intercept	-	-	-7.69	-
Area 2010	-	-	-	-
Forest_amount 2010_1	-	-	-	-
Forest_amount 1850_1	-	-	0.47	-
STW	-	-	-	-
CWD	-	-	-	-
ITS	-	-	-	-
Histo_continuity	-	-	-	-
Random effect (variance component)				
Zone	1.41	367.7	0	0
R^2^ marginal	NA	NA	0.60	NA
R^2^ conditional	NA	NA	0.60	NA

One hundred and two *A*. *unguiculare* individuals were sampled in five forests. All of these forests were in the south zone. GLMM showed a significant random effect of the zone (Table 2, variance component of *zone* variable > 0). The species was found present in one recent forest and four ancient forests.

One hundred and ninety-six *D*. *clathrata* individuals were sampled in 19 forests. The two GLMM (at 0.5 or 1 km) showed no significant effect of the zone (Table 2, variance component of zone variable = 0). The distribution of this species was only explained by *model 1*. In this model, Forest_amount 1850_1 was the only significant variable. The selected model, constituted by Forest_amount 1850_1 and random effect of the zone, had a marginal and conditional R^2^ of 0.60 (Table 2).

With 13 individuals in seven forests, *L*. *anophtalma* was the least abundant species in this study. The distribution of *L*. *anophtalma* could not be explained by the spatial, historical and structural forest variables used. Neither model 1 nor model 0.5 had significant variable (with a 95% confidence interval)(Table 2).

## Discussion

It was originally assumed that historical forest continuity was the main factor to explain the presence of the FLSB, modulated by the amount of habitat in the surrounding landscape. This hypothesis was not confirmed by the results of the present study. Historical forest continuity was not selected in the model.

### Species’ traits

*A*. *misellus* Boheman, 1844. [36] reports that this species has been trapped in the litter of several ligneous European genera, including *Buxus*, *Crataegus*, *Quercus*, *Betula*, *Tillia*, *Fagus* and *Abies*. As the diet of all known *Acalles* sp. is saproxylic [36,40,41], it was assumed that *A*. *misellus* is also saproxylic, and probably develops in thin branches like *A. parvulus [41]*.

*A*. *unguiculare* (Aubé, 1850). The adult lives in stumps, moss and leaf litter [41]. This species has a detritivorous diet and is considered to be a “facultative saproxylic” [40].

*D*. *clathrata* (Mannerheim, 1844). This species is saproxylic [42], as it has been reared on dead *Fagus* branches [43].

*L*. *anophtalma* Aubé, 1842. This species is saproxylic but has been caught in very different habitats, from coarse woody debris in forests to cellars [40,44].

None of these species were within the limit of their area of distribution and they were caught in northern, southern, eastern and western areas of the studied forest[14,22].

### The presence of species were poorly explained by dead wood resources and area

According to the literature, the volume of dead wood does not appear to explain either the species distribution or species richness of FSLB [21,22]. In line with the literature, the present study showed that the volume of dead wood had no effect on the presence of *D*. *clathrata*. The small size of the specie studied here led to the conclusion that they develop in thin branches or roots [20], which is generally not a limiting factor in harvested forests, and may explain the limited importance of the volume of available dead wood in the models.

The principle of island biogeography [28] led to expectations of higher species richness in larger forested areas, especially because larger forests have a higher probability of comprising diversified habitats [45]. With only five species, no analysis could be undertaken on species richness. Individually, it was expected that the extent of the forest would play a positive role in the presence of the species but in contrast to this hypothesis, the current area was not selected in the model.

There are two hypotheses that could explain this result. First, if the species does develop in thin branches and does not depend on a small number of particular tree species, then the resources in the habitat may not be a limiting factor, even in a small harvested forest. This is consistent with their limited dependence on dead wood resources mentioned above and in the literature [21,22]. Secondly, it is possible that the range of forest area was not sufficient to be able to evaluate its impact on species (maximum 82 ha).

### The amount of forest habitat in the surrounding area makes an important contribution to the species’ presence

Some studies have already shown that the occurrence of dispersal-limited species, such as herbaceous species, is determined by current but also past landscape composition [46,47]. To the best of the authors’ knowledge, the impact of forest amount on FSLB has only been taken into account in a highly forested landscape and no effect identified [21]. In the present study, past habitat amounts in the surrounding landscape were the only variables explaining the presence of *D*. *clathrata*.

The presence of *A*. *misellus* and *A*. *unguiculare* was significantly different in the three zones. *A*. *misellus* was found more in the south zone and *A*. *unguiculare* was only found in this zone, whereas these species are not within the limit of their area of distribution. The only variable that was significantly different between these zones was the forest amount at 0.5 km in 2010, which was higher in the south zone. Currently, the only explanation for the distribution of these two species is that they would at least be dependent on a high forest amount around the forest sampled. The presence of *A*. *misellus* in the west and east zone was in accordance with this explanation since the forests where the species were present had high forest amount in 2010 within 0.5 km radius.

The importance of past and current forest amounts in the surrounding area is in contrast with the recent results of [21], in which no impact of forest amount on FSLB was reported. This could be explained by: (1) the differences in species, even if they are all FSLB, and (2) the difference in forest amount surrounding the sampled plots. The study of [21] was conducted in a highly forested zone in the French Alps. In the present study, the landscape was a highly fragmented agricultural plain. The authors believe that the difference in the amount of forest in the landscape (63% vs. 19% in the present study) could explain the differences between the two studies: as the amount of forest in a landscape decreases, this factor becomes increasingly limiting.

The importance of landscape variables in *D*. *clathrata*‘s selected model and the explanation of the presence of *A*.*misellus* and *A*. *unguiculare* supported the hypothesis that dispersal-limited species are affected by the forest amount in the surrounding area (past and current) [46,47], especially when the landscape is very fragmented.

### The distribution of species appeared to be independent of forest temporal continuity

In highly forested landscapes, flightless saproxylic beetles appear to be able to recolonise recent forest [21,24]. In contrast, in highly fragmented landscapes such as those in north-west Germany, this group of species was reported to be present in ancient forest only [23].

The landscape in the present study was very fragmented, which is why temporal continuity was expected to be the main factor explaining species distribution. In contrast to this hypothesis, however, this variable was not selected in *D*. *clathrata’s* model (Table 2) and *A*. *unguiculare* and *A*. *misellus* were present in ancient and recent forest.

The absence of the selection of temporal continuity in the models could be explained by the fact that 1850 may not be a determining date for these species. In order to really be able to confirm this hypothesis, other periods before and after 1850 would need to be tested.

### *D*. *clathrata*: An indicator of past amount of forest in the landscape?

**Fig 3:**
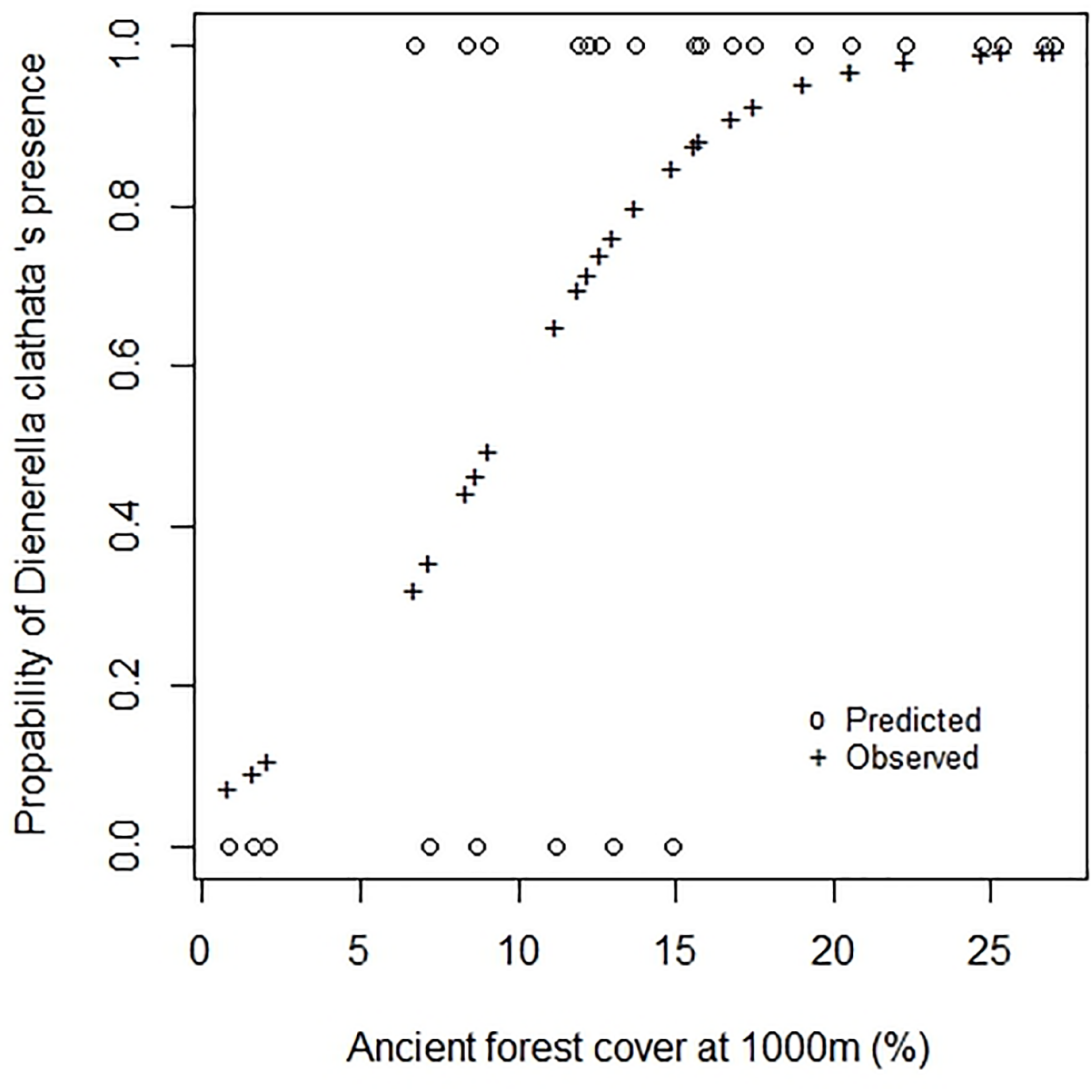
Comparison between the observed and predicted distribution of *D*. *clathrata* (model 1.0).

The *D*. *clathrata* distribution model took only one variable into account to explain the presence of a species (R^2^ = 0.60 Table 2). This species was absent in all the forests that had less than 5% forest habitat in their surrounding area in 1850, whereas the species was always present in forests that had more than 15% (Fig 3). The sigmoidal trend of the species distribution in Fig 2 suggests that the current presence of *D*. *clathrata* in one forest may be an indicator of the past amount of habitat in the surrounding of the forest concerned.

The potential indicator value of this species was tested with INDVAL [48]. Two groups of forests were defined: one in which the past amount of forest in the surrounding area was less than 15%, and the other where it was more than 15%. The indicator value of *D*. *clathrata* for the group >15% was 72.6% (9999 permutations, p-value = 0.0013). This value was composed of a fidelity value of 1 and a specificity value of 0.726. The threshold currently used to determine an indicator species is 25% [48,49]. With an INDVAL of 72.6%, *D*. *clathrata* may be considered to be an indicator species of forest amounts greater than 15% in 1850 in south-west France. To confirm this finding, additional studies should be carried out on this species and on the *Dienerella* genus. It is interesting to note that the threshold of 15% of forest in the landscape is close to the 20% to 30% of suitable habitat needed to maintain species [5].

### Species diversity in a highly fragmented landscape

At least 13 species of FSLB are present in the Grésigne forest, a biodiversity hotspot in south-west France [14]. As the Grésigne forest is located 100 km from the present study site and contains the same tree species, the 13 species of FLSB are considered to represent a potential pool for the species in the present study. However, only five species have been caught here to date.

In contrast to the Grésigne forest, the landscape in the present study area has been intensively deforested and fragmented for several centuries [31,50]. This major difference in human impact could explain the difference in species diversity between the biodiversity hotspot and the present study area. It is therefore possible that the five species remaining in this highly impacted landscape are the most mobile and resilient and/or whose diet does not depend on too limited a habitat.

## Flightless species: A different model of saproxylic beetles

The distribution of flying and/or large saproxylic beetle species is mainly driven by dead wood resources [18,21]. In a fragmented context, temporal continuity can also explain their diversity [30]. In this study and others [21,22,51], however, FSLB appeared not to be influenced at all (or only very slightly) by these parameters (dead wood resources and temporal continuity). FSLB therefore constitute a different biological model about which there is a considerable lack of knowledge and on which more studies need to be undertaken.

## Conclusions

The distribution of flightless saproxylic beetles was studied in a highly fragmented landscape. Past and current forest amount variables were shown or were assumed to play a greater role in the species distribution models than forest variables. Very few studies have been conducted to date to understand the distribution pattern of FSLB, yet this study underlines the potential interest of these species. Their presence on two dates was explored in a very fragmented landscape in which residual fauna are quite rare. More studies of the different taxa on other dates are needed in order to evaluate the impact of past landscape characteristics and to adapt future landscape management accordingly [27,46,47].

Species that are considered to be dispersal-limited such as herbaceous or flightless insects, thus appear to be more greatly influenced by the landscape characteristic than the forest characteristic [46,47]. This leads to the question of whether the way in which dispersal limitation is viewed is correct and weither the species can compensate for certain morphological contraints in order to disperse correctly. What kind of corridor do they use? Are they able to pass through or live in hedges? How long can an individual survive or how many generations can survive in isolated trees?
